# Supplementary figures and images for: Modelling the brain response to arbitrary visual stimulation patterns for a flexible high-speed Brain-Computer Interface
Source: PLoS One. 2018 Oct 22;13(10):e0206107. doi: 10.1371/journal.pone.0206107 (PMC6197660; doi:10.1371/journal.pone.0206107)

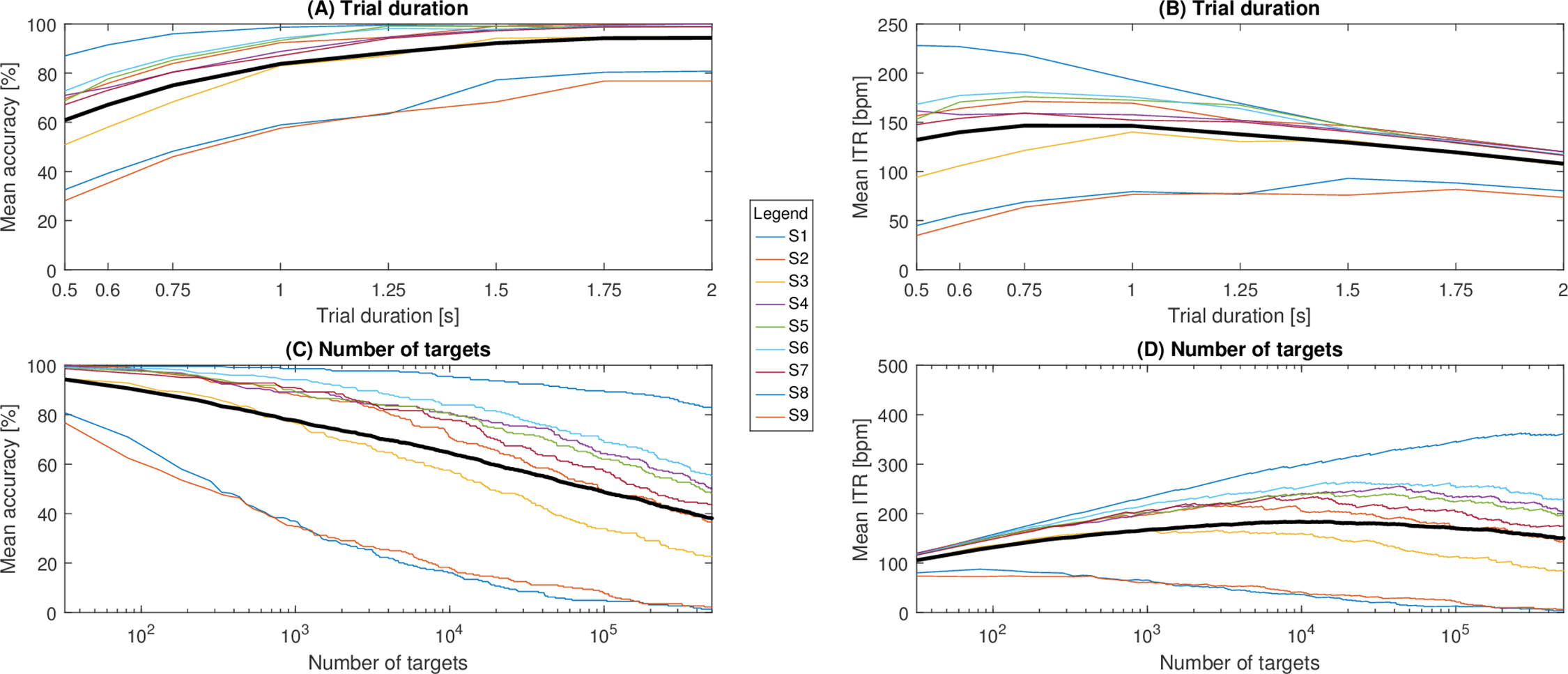

Supplement: S1 Fig — Shown are the accuracies and ITRs (including 0.5 s inter-trial time). Each colored line is one subject and the thick black line represents the mean of all subjects. A and B: using varying trial durations. C and D: using varying number of targets (logarithmic scale). (TIF) [file pone.0206107.s001.tif]

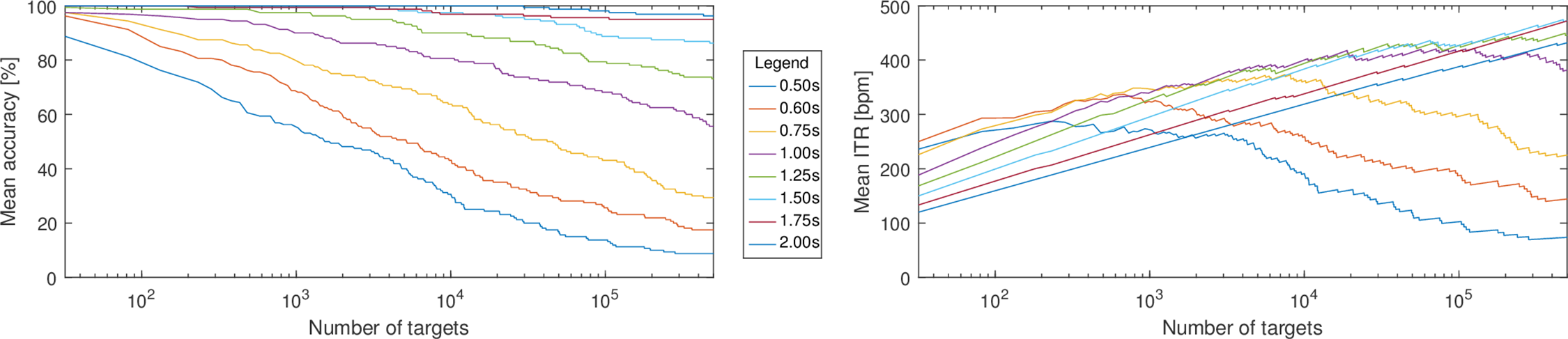

Supplement: S2 Fig — Shown are the accuracies and ITRs (including 0.5 s inter-trial time) using varying number of targets for varying trial durations. Each colored line represents a different trial duration. The maximum ITR of 474.5 bpm is reached using a trial duration of 1.5 s and 472,700 additional targets. (TIF) [file pone.0206107.s002.tif]

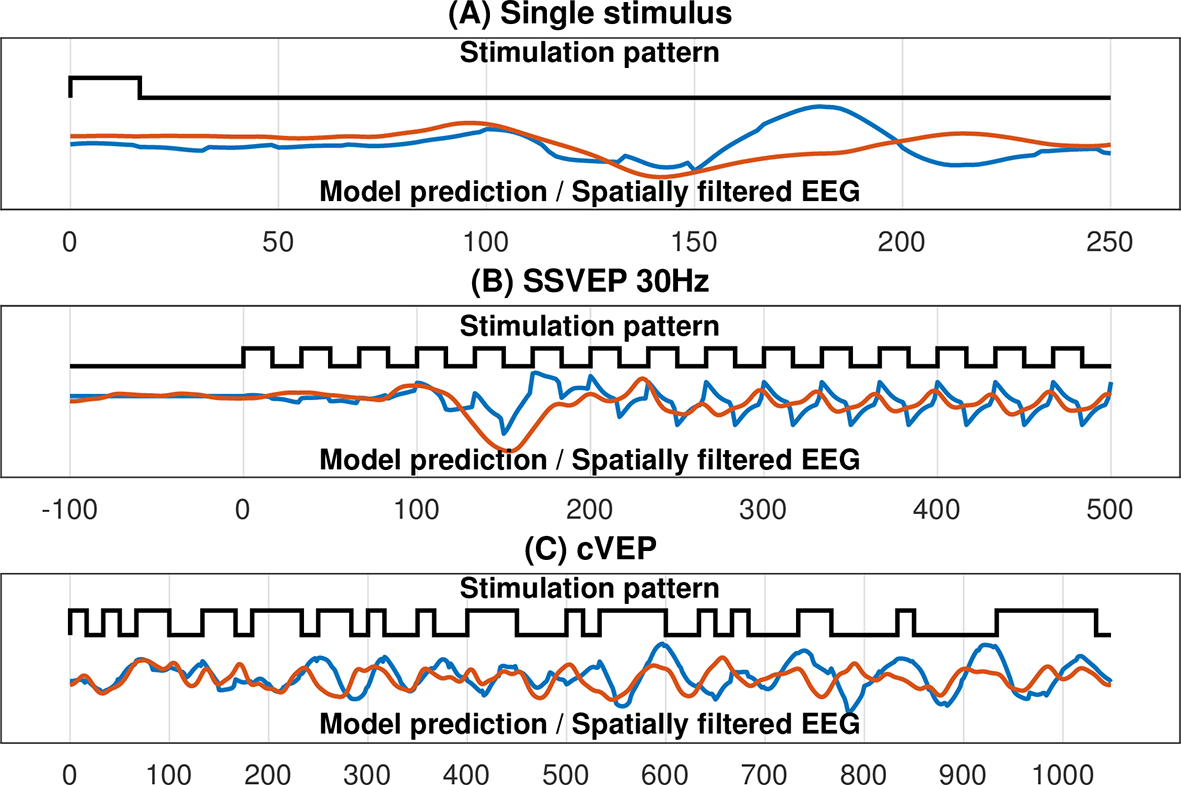

Supplement: S3 Fig — For the training, only stimulation patterns with < 3 bitchanges are used. The black line represents the stimulation pattern, the blue line the predicted brain response and the red line the spatially filtered EEG (120 trials averaged). A: Single stimulus pattern lasting for 1/60 s. B: 30 Hz SSVEP pattern. C: cVEP pattern. (TIF) [file pone.0206107.s003.tif]
